# Evaluating the utility of chest x-rays for non-traumatic chest pain in Australia- a retrospective cohort study

**DOI:** 10.1007/s10140-025-02329-2

**Published:** 2025-03-15

**Authors:** Alex Lin, Dinesh Varma, Biswadev Mitra

**Affiliations:** 1 School of Public Health & Preventive Medicine, Monash University, Melbourne, VIC 3004 Australia; 2 Emergency & Trauma Centre, The Alfred Hospital, Melbourne, Australia; 3 Department of Radiology, The Alfred Hospital, Melbourne, Australia; 4 Department of Surgery, Monash University, Melbourne, Australia

**Keywords:** Emergency medicine, X-Rays, Radiology

## Abstract

**Purpose:**

The aim of this study was to quantify the proportion of chest x-rays (CXRs) for non-traumatic chest pain (NTCP) in the emergency department (ED) that were abnormal and assess the clinical significance of these abnormalities. We also aimed to explore the variables associated with abnormal and clinically significant abnormal CXRs, to predict a population where CXRs can be safely avoided.

**Methods:**

A single center retrospective cohort study was conducted including all adult patients presenting to a single ED with NTCP between 01 Jan 2022 and 31 Dec 2022. We categorized the CXRs into abnormal, or normal as reported by a radiologist. Abnormalities were categorized to be clinically significant based on potential or actual changes in patient management. The association of patient demographics, presenting vital signs, and clinical characteristics with clinically significant abnormalities were explored using multivariable logistic regression analysis.

**Results:**

There were 3,419 eligible patient encounters included for analysis. Of these, 746 (21.8%; 95%CI: 20.4-23.2%) CXRs had at least one abnormality detected. There were 218 (6.4%; 95%CI: 6.1-7.9%) CXRs deemed to have clinically significant abnormalities. Age categories of 50–64 years (aOR 1.64; 95%CI 1.04–2.60), and age > 64 years (aOR 2.32; 95%CI: 1.51–3.57), history of congestive heart failure (CHF) (aOR 1.86; 95%CI: 1.08–3.21), smoking (aOR 1.27; 95%CI: 1.04–1.57), hemoptysis (aOR 6.69; 95%CI: 1.92–23.33), diminished lung sounds (aOR 4.87; 95%CI:2.95–8.05), rales (aOR 4.49; 95%CI: 2.82–7.15), and abnormal oxygen saturations (aOR 1.98; 95%CI: 1.40–2.79) were associated with clinically significant abnormalities on CXRs. In the absence of these variables, 1.4% (95%CI: 0.6-2.6%) of CXRs were abnormal with clinical significance.

**Conclusions:**

CXRs have a relatively high yield of abnormalities among patients with NTCP. However, some CXRs could be safely avoided in the absence of variables associated with clinically significant abnormalities. Further validation of these clinical characteristics is required before translation to clinical practice.

**Graphical Abstract:**

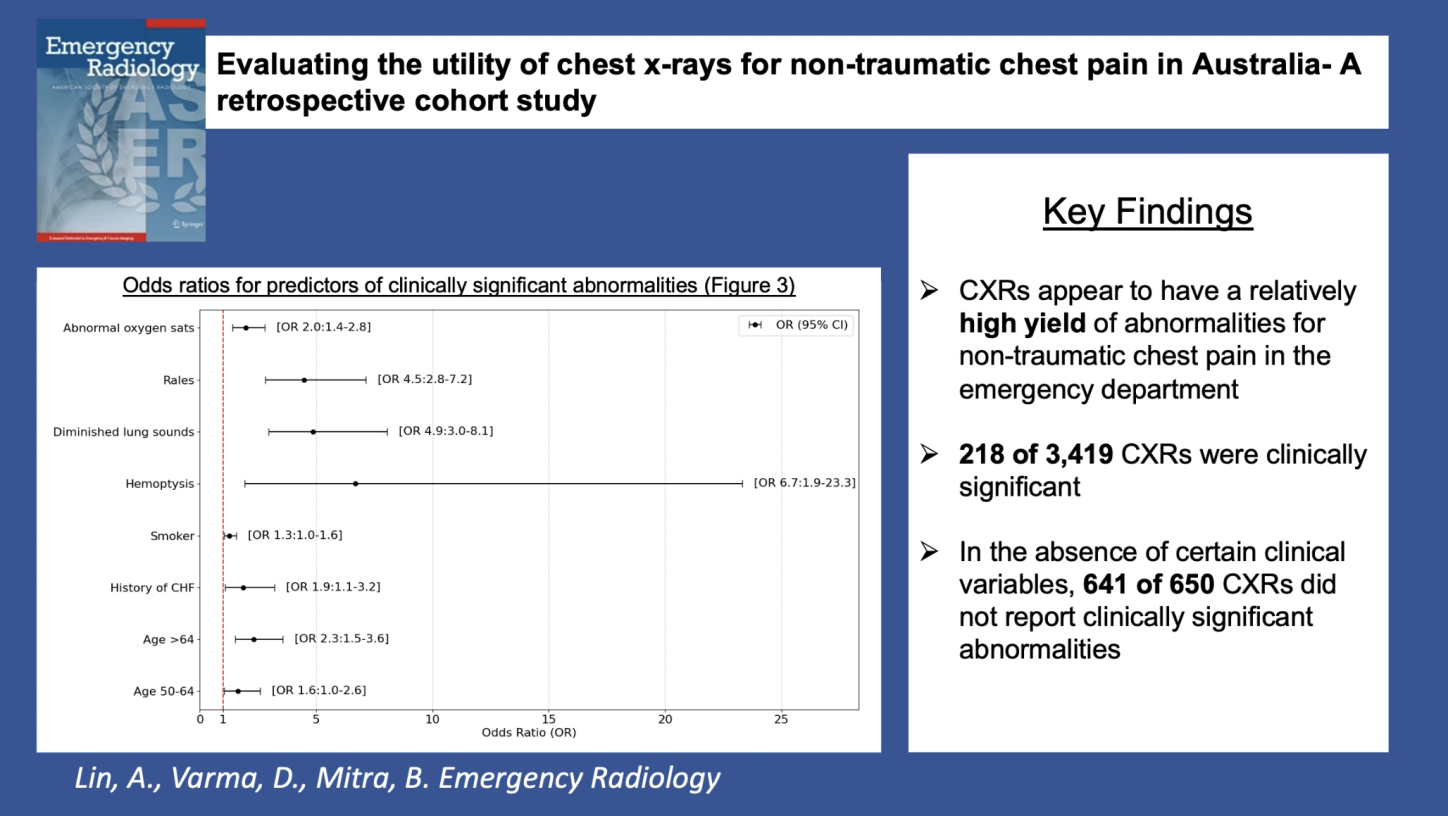

## Introduction

Chest pain is a common symptom, with 20–40% of the general population experiencing some form of chest pain in their lifetime [1]. In Australia, pain in the throat and chest ranks first in several emergency department (ED) presentations, with around 500,000 presentations of chest pain out of about 8 million ED visits a year [2, 3]. In addition, given the potentially fatal consequences of missing the diagnosis of acute heart, lung, or great vessel disease, chest pain is a presenting complaint generally considered to be of critical importance [4]. In most cases, the assessment of chest pain in the ED aims to rule out serious causes.

The use of a CXR as an investigation for chest pain is usually appropriate when guided by clinician judgment, according to the American College of Radiology (ACR) guidelines [5–7]. However, unlike traumatic injuries where the use of a CXR can locate fractures or diagnose pneumothoraxes, among patients with non-traumatic chest pain (NTCP), CXRs have lower sensitivities for other pathology [5, 7]. While CXRs can demonstrate pneumonia or signs of heart failure, sensitivities of CXR for pneumonia or acute decompensated heart failure are moderate, at best [8, 9]. 

Liberal use of CXRs for NTCP could be detrimental to patients through unnecessary radiation exposure, and even though a CXR only takes a few minutes to perform, this does not include the time taken to request a CXR, organize patient transport, review, and report images. The combined times for requisition, performing and reporting of CXRs can create both a delay in diagnosis and an increased length of stay in the ED, contributing to overcrowded EDs [10, 11]. 

In addition, the sustainability of future healthcare funding has been a growing concern, with predictions of healthcare expenditure as a percentage of gross domestic product doubling in 2050 [12]. It is therefore important that healthcare resources remain directed more towards addressing major issues, and that we reduce unnecessary expenditures. An Australian cost-effectiveness study by Cullen et al. [13] reported that the mean ED stay for chest pain suggestive of acute coronary syndrome in the ED was AU$162 per hour, compared to the average estimate of AU$98 per hour for all ED complaints. This difference was attributed to the high triage category of chest pain and the investigations involved in assessing chest pain, such as CXRs.

The aim of this study was to quantify abnormalities on CXRs for NTCP in the ED, and also to quantify abnormalities of clinical significance. The overarching aim was to identify a population in whom, with the absence of risk factors for clinically significant abnormalities, CXRs could be safely avoided.

## Methods

### Setting

The study was conducted in an adult major referral hospital in metropolitan Melbourne, Victoria, with an annual emergency department (ED) attendance of approximately 65,000 patients. The hospital provides state services for major trauma, burns, hemophilia, cystic fibrosis, heart and lung transplants, HIV, and adult hematological malignancies.

Design: A retrospective cohort study was conducted using explicit chart review.

### Inclusion criteria

We included all adult patients who were investigated with a CXR in the ED between 01 Jan 2022 and 31 Dec 2022.

### Exclusion criteria

The exclusion criteria consisted of patients who were under 18 years old, patients with more than one CXR during their presentation, patients without chest pain, patients with a traumatic etiology of chest pain, and patients without any medical history documented.

### Data extraction

Data were extracted from the electronic medical record system using explicit chart review by a single investigator (AL). Data on demographics, presenting vital signs, hemoptysis, chest auscultation findings (diminished lung sounds, rales, wheezes), and history of venous thromboembolic disease (VTE), chronic lung diseases (including chronic obstructive pulmonary disease, asthma, cystic fibrosis, idiopathic pulmonary fibrosis, interstitial lung disease, history of lung transplant), heart failure, and smoking were extracted.

Past medical history and chest auscultation findings were categorized into present or absent. Smoking history was categorized as non-smoker, ex-smoker, or current smoker. Vital signs were categorized into abnormal or normal, based on the Royal Prince Alfred Hospital patient observation vital signs policy [14]. A respiratory rate of 12–20 breaths per minute, heart rate of 60–100 beats per minute, systolic blood pressure of 90–129 mmHg, temperature of 36.5-37.2^o^C, and oxygen saturations of 97–100% were considered normal [14]. 

Radiology reports were extracted and CXRs were subgrouped into those with abnormalities reported and compared to those without. CXRs with abnormalities were subgrouped into those that were clinically significant and compared to those that were not. Overt signs of heart failure, consolidation, masses, and fractures that were likely to change management were considered to be clinically significant. Where signs of heart failure or infection were reported to be subtle, medical records were further reviewed to extract data on whether the abnormality had changed the clinical management of the patients. Specifically, the initiation of acute heart failure therapy or antibiotic therapy in the ED was considered to be clinically significant.

### Statistical analysis

All data were summarized using counts (proportions) and differences were assessed using the chi-square test. The association of abnormal and clinically significant abnormal CXRs with clinical characteristics were assessed using univariable logistic regression analysis. Following this, a multivariable logistic regression model was developed with backward elimination. Results were reported using odds ratios and adjusted odds ratios with 95% confidence intervals. A p-value of < 0.05 was defined to be statistically significant.

Post-estimation testing was performed for the multivariable regression model using the Hosmer-Lemeshow test and variance inflation factor (VIF). The Hosmer Lemeshow test identifies the goodness of fit of the collected data to the model, and the VIF measures the levels of multicollinearity within the model.

All analyses were performed using Stata v18.0, College Station, TX, USA.

### Ethics

The study was approved by the Alfred Hospital Human Research Ethics Committee. The requirement to seek informed consent from patients was waived by the ethics committee.

## Results

**Fig. 1:**
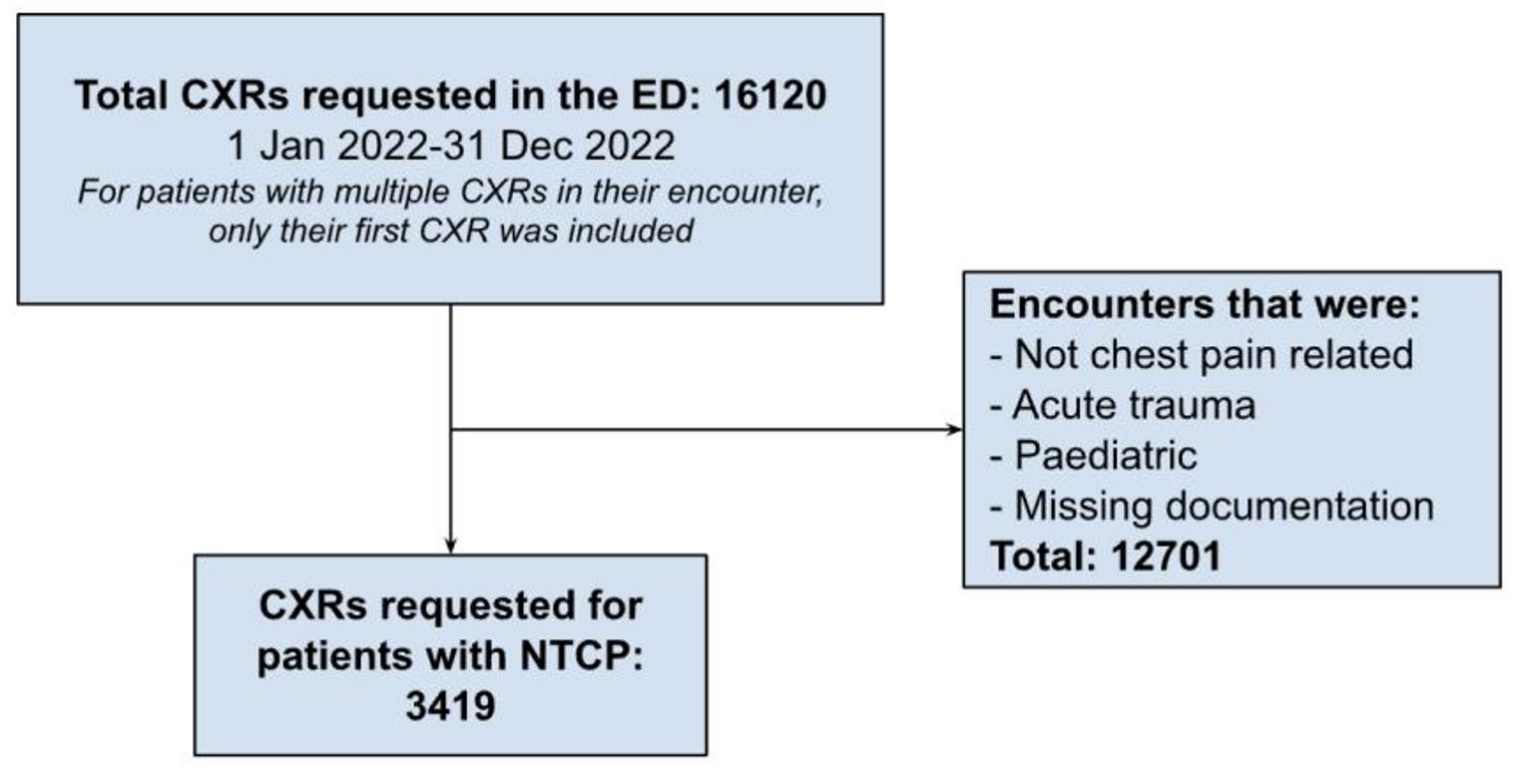
Flowchart of the filtration of total CXRs in the ED to CXRs requested for patients presenting with NTCP

There were 71,606 patient encounters identified in the ED during the study period. Of these encounters, 16,120 were investigated with a CXR in the ED. After excluding patients with non-chest pain-related causes, traumatic chest pain, missing documentation, and patients who were pediatric, 3,419 patient encounters with CXRs and NTCP were included for analysis. Figure 1 displays the selection of patients.

Among included patients, there were 746 (21.8%; 95%CI: 20.4–23.2) CXRs with an abnormality detected.

**Fig. 2:**
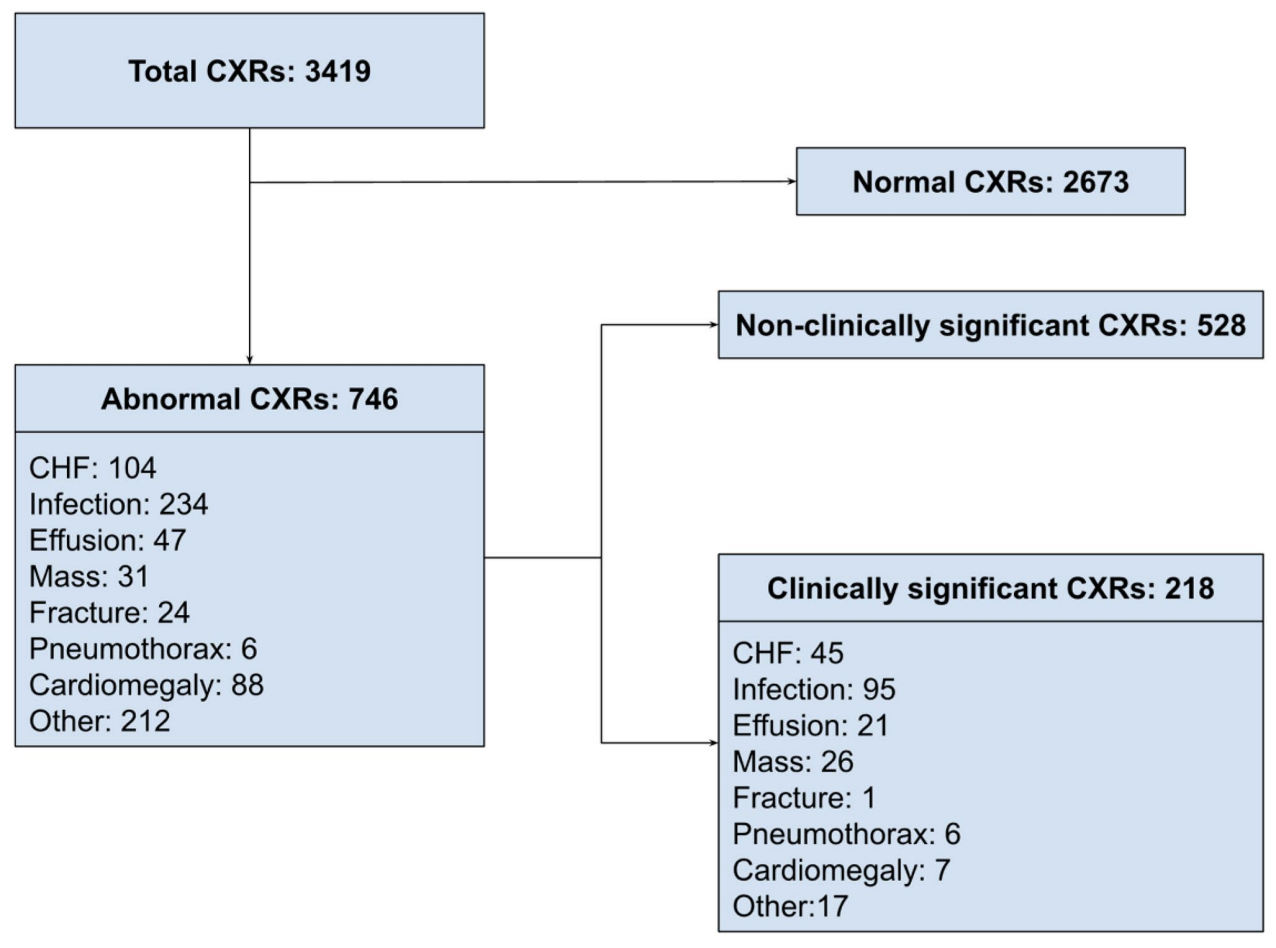
Categorization of abnormalities and clinically significant abnormalities on CXR; CXR: Chest X-Ray, CHF: Congestive heart failure

The most common abnormality category was features of an infection (*n* = 234; 6.8% of all CXRs). Other abnormalities seen were CHF (*n* = 104), effusion (*n* = 47), mass (*n* = 31), fracture (*n* = 24), pneumothorax (*n* = 6), cardiomegaly (*n* = 88), and other (*n* = 212). The abnormalities were categorized in Fig. 2.

There were 218 (6.4%; 95%CI: 6.06–7.87) CXRs that were deemed to have clinically significant abnormalities. The most common clinically significant abnormality was infection (*n* = 95; 2.8% of all CXRs). Other clinically significant abnormalities were findings of CHF (*n* = 45), effusion (*n* = 21), mass (*n* = 26), fracture (*n* = 1), pneumothorax (*n* = 6), cardiomegaly (*n* = 7), and other (e.g. aortic dissection, large hiatus hernia, pseudomediastinum, *n* = 17; Fig. 2). The univariable association of variables associated with clinically significant abnormal CXRs was listed in Table 1.

**Table 1:** Univariable association of clinical variables with clinically significant abnormal CXRs

Variable	OR (95%CI)	*p*-value
Age (years)		
• < 50	Ref	
• 50–64	1.67 (1.13–2.46)	< 0.001
• > 64	3.52 (2.55–4.86)	< 0.001
Male sex	1.17 (0.89–1.55)	0.25
History of VTE	1.30 (0.71–2.39)	0.40
Chronic lung disease	2.31 (1.67–3.19)	< 0.001
History of CHF	6.02 (3.93–9.20)	< 0.001
Smoker	1.28 (1.09–1.51)	0.003
Hemoptysis	4.96 (1.59–15.50)	0.006
Diminished lung sounds	8.24 (5.32–12.77)	< 0.001
Rales	9.37 (6.29–13.95)	< 0.001
Wheeze	3.43 (1.80–6.52)	< 0.001
Abnormal HR	1.42 (1.04–1.95)	0.029
Abnormal RR	2.87 (2.04–4.04)	< 0.001
Abnormal SBP	0.87 (0.66–1.15)	0.27
Abnormal Temperature	0.81 (0.61–1.09)	0.16
Abnormal oxygen saturations	3.25 (2.46–4.30)	< 0.001

**Fig. 3:**
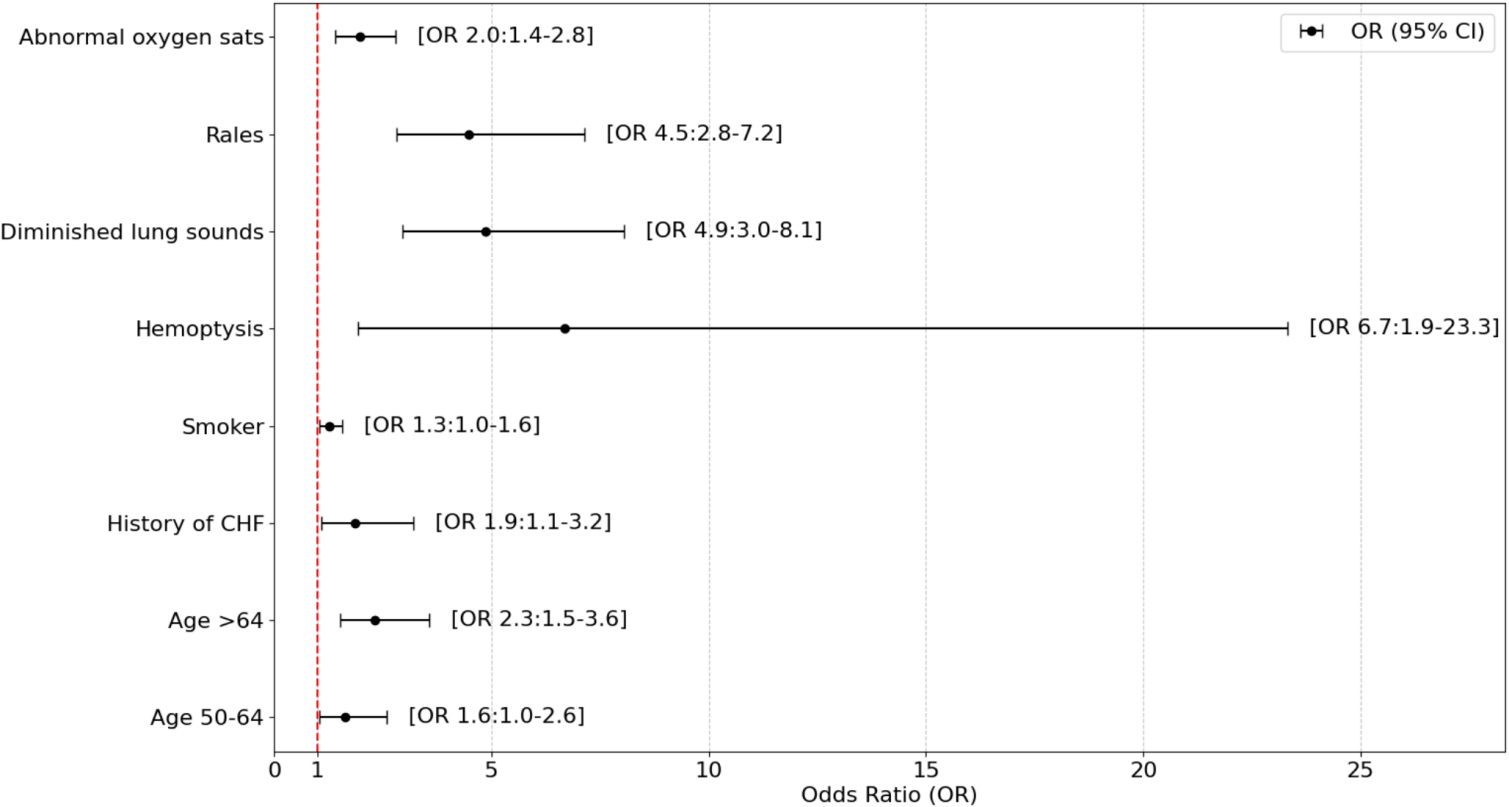
Odds ratios for predictors of clinically significant abnormalities

Following multivariable regression and backward elimination, older age, history of CHF, smoking, hemoptysis on presentation, diminished lung sounds, rales, and abnormal oxygen saturations were independently associated with clinically significant abnormalities on a CXR. These variables were listed in Table 2, with the odds ratios for each variable illustrated in Fig. 3.

**Table 2:** Multivariable regression of clinical variables with clinically significant abnormalities

Variable	OR (95%CI)	*p*-value
Age (years)		
• < 50	Ref	
• 50–64	1.64 (1.04–2.60)	< 0.001
• > 64	2.32 (1.51–3.57)	< 0.001
History of CHF	1.86 (1.08–3.21)	0.024
Smoker	1.27 (1.04–1.57)	0.019
Hemoptysis	6.69 (1.92–23.33)	0.003
Diminished lung sounds	4.87 (2.95–8.05)	< 0.001
Rales	4.49 (2.82–7.15)	< 0.001
Abnormal oxygen sats	1.98 (1.40–2.79)	< 0.001

For the final model predicting clinically significant abnormal CXRs, the p-value for the Hosmer-Lemeshow test for the final model was 0.03. The mean-variance inflation factor (VIF) was 1.12, with maximum VIF of 1.33.

The clinical decision rule was defined as the absence of statistically significant variables associated with clinically significant abnormal CXRs. The summary statistics describing the sensitivity, specificity, and predictive values of this clinical decision rule for predicting clinically significant abnormal CXR in our population were listed below in Table 3. In the absence of the statistically significant variables associated with abnormalities, there were 9 (1.4%) clinically significant abnormalities remaining. These abnormalities were listed in Supplementary Table 1.

**Table 3:** Performance of decision rule to predict clinically significant abnormal CXRs

	Result	95% Confidence interval
**Sensitivity**	95%	90.8-97.7%
**Specificity**	26.8%	25.0-28.6%
**Positive Predictive Value**	8.9%	7.7-10.3%
**Negative Predictive Value**	98.6%	97.4-99.4%

## Discussion

Among patients investigated with CXR for non-traumatic chest pain, a high rate of abnormalities (21.8%) and clinically significant abnormalities (6.4%) were detected. Younger age, with the absence of CHF, hemoptysis, a history of smoking, together with clear auscultation and normal oxygen saturations had a 99% sensitivity to rule out clinically significant abnormal CXRs. In this population without the above clinical features, 9/650 (1.4%) had clinically significant abnormal CXRs. Thus, CXRs for NTCP in this population should be reconsidered.

These findings were consistent with previous literature, which reported varying rates of clinically significant abnormal CXRs from 2-17%. [15] Our predictive criteria showed similarities to that of Newsom et al. [15], which combined criteria from Rothrock et al. [16] and Hess et al. [17]. However, our study did not find history of venous thromboembolism nor fever to be associated with clinically significant abnormal CXRs. We excluded history of alcohol abuse as a variable as it was difficult to ascertain retrospectively, and also excluded history of tuberculosis given its low prevalence in our study population.

The prevalence of clinically significant abnormal CXRs after accounting for missing data in the predictive criteria was 7%, similar to previous studies on the topic [15]. An age of at least 50 years old was found to be associated with clinically significant abnormal CXRs. While the aging process is not fully understood, it has been proposed that it involves the malfunctioning of cellular and molecular events, which can be linked to the development of various chronic diseases [18]. With age, also comes the long-term exposure to many other confirmed risk factors for disease, such as tobacco, vaping, alcohol, stress, and lack of exercise [18]. In this way, age becomes a major risk factor for chronic diseases like heart failure, which could produce abnormal findings of cardiomegaly and effusions on CXRs [19]. With older age also comes remodeling of the immune system, causing immune senescence and making patients more susceptible to infection and cancers [20]. 

Findings of hemoptysis in the context of NTCP can be associated with pneumonia, lung abscess, cancer, or pulmonary embolism [21]. Auscultation findings of diminished lung sounds can stem from either weak sound generation or impaired transmission from obstruction. Possible causes are obstructive respiratory conditions like asthma and COPD, pleural effusion, pneumothorax, or shallow breathing upon examination. Our definition of rales included coarse and fine crackles or crepitations, with fine crackles being heard in conditions like early congestive heart failure and interstitial lung disease. Coarse crackles are heard in conditions like asthma, COPD, pneumonia, and pulmonary edema [22]. However, even though the auscultation findings were found to be associated with clinically significant abnormal CXRs, they are highly variable as clinical features to utilize. Auscultation is subject to differing clinical expertise, external noise level, and patient factors like body habitus, or effort of breathing during the examination [23]. Oxygen saturations are in comparison more reliable as a clinical marker for disease [24]. 

## Limitations

This was a retrospective study with its inherent biases and relied on contemporaneous documentation in medical records. Prospective validation would help determine the rule’s effectiveness in real-world ED environments, especially given its low specificity. Compared with previous similar studies, where Hess et al. [17], and Poku et al. [25] defined a specific population (patients at high risk for ACS), we included all patients who presented with non-traumatic chest pain. Therefore, higher sensitivities may be possible among sub-groups of patients.

All data were collected by a single investigator, and even though it was done objectively, there could be some measurement bias involved. Data were collected from a major tertiary hospital in a metropolitan area, which could be less representative of the general population and not capture the more remote and rural areas with a different burden of disease.

As our study was conducted on a patient population from 2022, COVID-19 was a present factor. The sensitivity of a CXR for COVID-19 was 56–69% [26, 27], similar to the sensitivities of detecting congestive heart failure and pneumonia, which hover around the 50–80% mark [8, 28, 29]. It is possible COVID-19 could have impacted our study by increasing the proportion of abnormalities on CXRs compared with pre-COVID cohorts.

## Conclusion

The application of certain demographics and findings on history and examination to patients presenting with non-traumatic chest pain to the emergency department may be overall beneficial in reducing the number of CXRs performed. Multi-center studies with diverse populations are indicated to validate the results of this study.

## Appendix

*Supplementary Tables 1- Clinically significant abnormalities missed by the clinical decision rule*.

**Table:** 

Clinically significant abnormalities missed	Number of CXRs
Infection	3
Effusion	1
Mass	2
Fracture	1
Pneumothorax	1
Other	1

## Data Availability

The data that support the findings of this study are available from the corresponding author upon reasonable request and subject to ethics committee approval.
